# Utility and Limitation of Cumulative Stone Diameter in Predicting Urinary Stone Burden at Flexible Ureteroscopy with Holmium Laser Lithotripsy: A Single-Center Experience

**DOI:** 10.1371/journal.pone.0065060

**Published:** 2013-06-04

**Authors:** Hiroki Ito, Takashi Kawahara, Hideyuki Terao, Takehiko Ogawa, Masahiro Yao, Yoshinobu Kubota, Junichi Matsuzaki

**Affiliations:** 1 Department of Urology, Ohguchi East General Hospital, Yokohama City, Japan; 2 Department of Urology, Yokohama City University Graduate School of Medicine, Yokohama City, Japan; Universidade de Sao Paulo, Brazil

## Abstract

**Purpose:**

To retrospectively assess the clinical utility in ureteroscopy (URS) planning of cumulative stone diameter (CSD), which does not account for stone width or depth, as a predictor of URS outcome and compare it with stone volume.

**Materials and Methods:**

Patients with renal stones treated at a single institute by flexible URS were retrospectively evaluated. To assess the clinical utility of CSD, relationships between stone-free (SF) status and stone burden (CSD and volume) were analyzed using the area under the receiver operating characteristics (AUROC) curve. To identify stone number impact on CSD, the AUROC of CSD divided by stone number was evaluated. Correlation coefficients of CSD and stone volume were also calculated for groups by stone number.

**Results:**

In cases with CSD <20.0 mm, CSD and stone volume revealed equal ability to predict SF status. In cases with CSD ≥20.0 mm, stone volume showed higher predictive ability. The ROC curves for cases with ≥4 stones showed that CSD was less predictive of SF status than stone volume. The correlation coefficients of CSD and stone volume by stone number were 0.922 for 1 stone, 0.900 for 2–3 stones, and 0.661 for ≥4 stones.

**Conclusions:**

In cases with CSD ≥20.0 mm or ≥4 stones, we should evaluate stone volume for a more predictive stone burden, and pretreatment non-contrast CT seems sufficient. In cases with CSD <20.0 mm or 1–3 stones, CSD was as valid a predictor of preoperative stone burden as stone volume, so preoperative kidney-ureter-bladder (KUB) films may be sufficient.

## Introduction

With the development of smaller caliber semirigid and flexible ureteroscopes, ureteroscopy (URS) has become a safer and more established modality for treating any type of urinary stone [1], [2]. Several reports have even described the safe and effective removal of multiple and large intrarenal stones with URS [3]–[5]. Thus, determining preoperative predictors of post-URS stone-free (SF) status remains crucial for maximizing the efficacy and safety of this procedure. In particular, assessment of a patient’s stone burden plays an important role in the management of their urinary stones [6]–[8]. However, there are no formal guidelines for the preoperative assessment of stone burden [8], [9].

Several stone parameters that reflect stone burden, including the cumulative diameter, surface area (SA), and volume, have been considered in URS studies [8], [9]. We previously examined the utility and priority of three parameters of renal stone burden (cumulative stone diameter [CSD], SA, and volume) at URS and found that stone volume determined by non-contrast computed tomography (NCCT) and CSD obtained by kidney-ureter-bladder (KUB) films were significantly and independently predictive of stone status after URS [8].

In clinical practice, the most widely used parameter is CSD [1]–[5]. In fact, The EAU and AUA Nephrolithiasis Guideline Panel used CSD as their measure of stone burden in when deciding on the treatment method for urinary stones [1], [2]. It is the simplest and most easily obtained among the parameters of stone burden. However, it does not factor in the stone width or depth, and therefore CSD as a predictor of URS outcome must be limited in comparison with stone volume. Nonetheless, the clinical utility and limitations of this parameter in predicting URS outcome have yet to be investigated. In this study, we retrospectively assessed the characteristics and clinical utility of CSD as a possible predictor of URS outcome.

## Materials and Methods

This study was approved by the Ethics Review Board of Ohguchi East General Hospital. All patients provided written informed consent for their data to be used for research purposes. The funders had no role in study design, data collection and analysis, decision to publish, or preparation of the manuscript.

### Patients

We retrospectively analyzed 314 flexible URS procedures for removal of renal stones, performed between October 2009 and October 2011 in Ohguchi East General Hospital. Of these 314 procedures, 71 were excluded from the study because of either a lack of analyzed parameters, completely radiolucent stones, unclear KUB films because of obesity or fecal artifacts, staghorn calculi, sponge kidney, or more than two stages of URS. The remaining 243 eligible procedures consisted of first-time URS or first-stage URS in multistage URS. The indications for urolithiasis treatment in our hospital are as follows: we generally recommend percutaneous nephrolithotripsy as the first-line treatment for renal stones >20 mm in diameter, and extracorporeal shockwave lithotripsy or medical expulsive therapy for urinary stones <10 mm in diameter. For all patients, URS was offered as a first- or second-line treatment. The final decision regarding the treatment was made by the patients themselves.

### Technique

Our surgical technique was well-described in our previous report [8], [10]. Briefly, Patients were given intravenous preoperative antibiotics. Before starting URS, the patient was placed in the dorsal lithotomy position under anesthesia. A 22.5 Fr cystoscope was inserted into the bladder through the urethra, allowing visualization of the ureteral orifice. This was cannulated with an open-ended 5 Fr catheter and a 0.038-inch hydrophilic guidewire. To directly visualize the intraureteral space, a 6/7.5 Fr or 8/9.8 Fr semirigid ureterorenoscope (Wolf™, Knittlingen, Germany) was passed over a guidewire using fluoroscopic guidance until it reached the proximal ureter or renal pelvis. Next, a ureteral access sheath (12/14 Fr or 14/16 Fr, Cook Medical, Bloomington, IN or 11/13 Fr or 13/15 Fr, Boston Scientific, Natick, MA) was placed, and flexible URS (Flex-X2™, STORZ, Germany or Olympus P-5™, Olympus, Tokyo, Japan) with 200-µm holmium laser lithotripsy was performed. If we failed to place a ureteral access sheath due to a tight ureter or ureteral stricture, a double-J stent was placed and a procedure was performed 2–6 weeks later. When basketing was deemed necessary, we used a 1.9 Fr zero-tip nitinol stone basket (Boston Scientific) or a 1.5 Fr N-Circle nitinol tipless basket (Cook Medical). A double-J stent was placed in all patients after endoscopy, and the bypass was removed 2–4 weeks postoperatively when we were certain that the bypass was no longer necessary.

### Clinical and Imaging Assessments

The preoperative factors analyzed included CSD (mm), stone volume (mm^3^), number of stones, stone side (right or left), age, sex, body mass index (BMI), presence of hydronephrosis and lower pole calculi, placement of the ureteral stent, and extracorporeal shockwave lithotripsy failure cases. The maximum diameter was measured on a plain KUB film. The stone volume was obtained from measurements on 3D images of the stone, using 5-mm axial and 3.5-mm reconstructed coronal NCCT. We used the following formula to calculate the stone volume: length × width × height × π × 1/6 [8], [10]. The maximum stone diameter on KUB films, as well as the stone length, width, and height on NCCT were determined using digital calipers (SYNAPSE-PACS Software Program System, Fujifilm, Tokyo, Japan) [8], [10], [11]. The presence of hydronephrosis (including even partial hydronephrosis) and lower pole calculi and the number of stones were also determined by preoperative NCCT.

A plain KUB film was obtained on postoperative day 1 in all cases to assess the presence of stones. The stone status–the primary outcome measure–was judged in all cases by the same investigator (H.I.). SF status was defined by no detectable stone on KUB films.

### Statistical Analysis

The data were analyzed using the SPSS software package (SPSS, Chicago, IL). At first, to assess the clinical utility of CSD, the relationships between SF status and the two parameters of stone burden (CSD and volume) were analyzed using the area under the ROC (AUROC) curve [12].

Subsequently, to identify the impact of stone number on CSD, the AUROC of CSD divided by stone number were also evaluated. Furthermore, the correlation coefficient of CSD and stone volume was calculated for 3 groups of patients based on stone count: 1, 2–3, and ≥4.

## Results

There were 243 eligible procedures. Of these, 236 were performed under general anesthesia and 7 under spinal anesthesia. A ureteral sheath was used in all 243 procedures. One major intraoperative complication occurred, a ureteral perforation resulting in retroperitoneal extravasation of urine in one patient. Minor complications after treatment included high-grade fever in 16 patients. All were treated conservatively. Long-term complications such as ureteral stricture formation occurred in 2 patients: one patient was treated with ureteral balloon dilation and has had a successful outcome, ureteral balloon dilation failure in the other patient was treated with placement of a permanent double-J stent.

**Figure 1:**
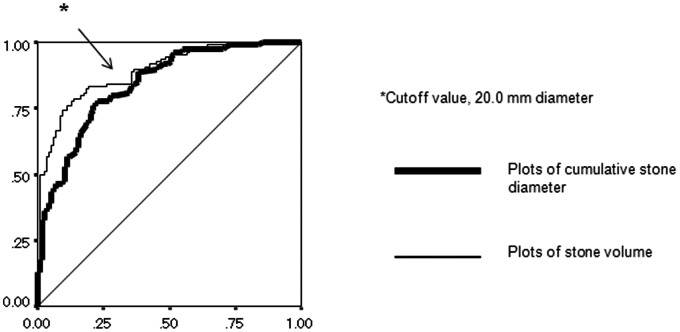
Receiver operating characteristics (ROC) curve of stone-free (SF) status on postoperative day 1 for the two parameters of stone burden, cumulative stone diameter and stone volume (n = 243).

The ROC curve of the two parameters of stone burden based on SF status after a single URS are shown in Fig. 1. The single cut-off value of predictive ability between CSD and stone volume was 20.00 mm. This suggests that CSD and stone volume are equally beneficial in predicting stone status after URS for patients with a maximum stone diameter <20.0 mm (Fig. 1). However, in patients with CSD ≥20.0 mm, CSD was inferior to stone volume as a predictor of SF status (Fig. 1). To assess the difference of backgrounds between groups with <20.0 mm diameter and ≥20.0 mm diameter, Table 1 shows a comparison of the patients’ demographic data and perioperative surgical outcomes according to stone diameter. There were significant differences between the <20.0 mm diameter and ≥20.0 mm diameter groups in the following parameters: number of stones (P<0.001), presence of lower pole calculi (P<0.001), diameter (P<0.001), volume (P<0.001), duration of procedure (P<0.001), amount of laser use (P<0.001), and SF rate on postoperative day 1 (P<0.001).

**Table 1:** Comparison of patient and stone data between cases with less than 20.0 mm stone diameter and more than 20.0 mm stone diameter.

		<20.0 mm	≥20.0 mm	*P*	
No. of patients (cases)		107	136		
Age (years)		60.56±13.41	59.05±13.13	NS	
Sex	female (cases)	49	55	NS	
	male (cases)	58	81		
BMI (cm/kg2)		22.87±4.80	23.01±5.60	NS	
Stone side	rt (cases)	50	58	NS	
	lt (cases)	57	78		
No. of stones		1.41±0.71	3.48±2.40	<0.001*	
	1	76	29	<0.001*	
	2,3	30	54		
	≥4	1	53		
intrarenal stone location	without lower pole calculi	54	31	<0.001**	
	with lower pole calculi	53	105		
diameter (mm)	mean ± SD	12.26±4.53	37.66±16.23	<0.001*	
	median (range)	11.50 (2.5–19.60)	32.95 (20.0–1104)		
volume (mm3)	mean ± SD	563.62±4.53	3080.56±3837.85	<0.001*	
	median (range)	419.12 (15.89–2329.60)	2024.38 (222.95–31275.38)		
Hydronephrosis (cases)		54	52	NS	
Preoperative stenting (cases)	deliberate prestenting	15	27	NS	
	stent due to pain or infection	36	56		
Duration of operation (min)		80.93±37.90	105.78±24.92	<0.001*	
Amount of laser use (J)		1.50±2.24	5.63±5.50	<0.001*	
Hospital stay (days)		3.66±2.07	3.75±1.94	NS	
Operator (No. of procedures)	experienced ≥50	73	96	NS	
	inexperienced <50	34	40		
ESWL failure (cases)		42	43	NS	
Stone-free rate at POD1 after URS		79.43% (85/107)	29.41% (40/136)	<0.001**	
Postoperative ureteral stricture (cases)		2	0		
Postoperative fever (cases)		8	8	NS	

*Mann-Whitney *U* test;

** Chi-square test;

NS: Not significant.

**Figure 2:**
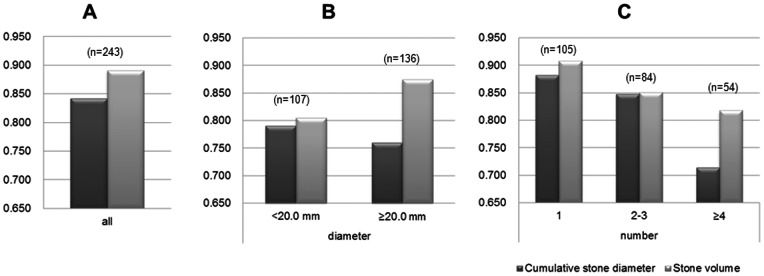
Value of the area under the receiver operating characteristic (AUROC) curve of cumulative stone diameter (CSD) and stone volume in all cases, divided by stone diameter (<20.0 mm/≥20.0 mm) and number (1, 2–3, ≥4).

The values for the AUROC curve [12] of the two parameters of stone burden are shown in Fig. 2. In all cases, these two parameters were highly indicative of SF status, and the correlation coefficient for CSD and stone volume was 0.835. In cases with CSD <20.0 mm, the two parameters demonstrated equal ability to predict SF status (Fig. 2). In cases with CSD ≥20.0 mm, however, stone volume showed higher predictive ability than the CSD (Fig. 2). The correlation coefficient for CSD and stone volume divided into stone number were 0.712 for patients with CSD <20.0 mm and 0.596 for patients with CSD ≥20.0 mm.

**Figure 3:**
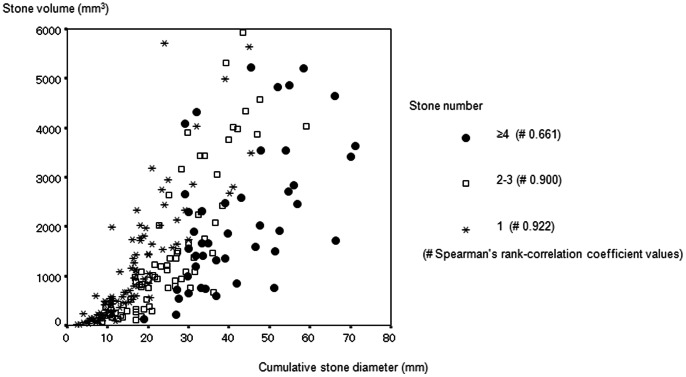
Scatter diagram using CSD and stone volume.

The ROC curves for cases with ≥4 stones showed that CSD was less predictive of SF status than stone volume (Fig. 2). The correlation coefficient for CSD and stone volume divided into stone number were 0.922 for patients with 1 stone, 0.900 for patients with 2–3 stones, and 0.661 for patients with ≥4 stones (Fig. 3).

## Discussion

We reported that, among the several parameters regarding the renal stone burden, the stone volume determined by NCCT and the CSD of the KUB were significantly and independently inversely related to the success rate of URS [8]. Several previous studies reported that NCCT had a higher potential to evaluate urinary stone than KUB [13]–[15]. In fact, in our previous study, the stone volume determined by NCCT is the strongest predictor of SF status after URS [8]. In clinical practice, however, the most common and well-established parameter of stone burden is CSD [1]–[5]. In addition, CSD is the simplest and most easily obtained parameter, so is the more available and efficient method. To resolve this discrepancy, this study examined the utility and limitations of CSD at URS compared with stone volume.

Based on the ROC curve of the two parameters of stone burden for SF status after a single URS (Fig. 1), the bigger the stone burden, the worse the predictive ability of CSD in comparison with stone volume. The ROC curve indicated that the cut-off point was near 20.0 mm (Fig. 1), which was consistent with our AUROC curve analysis (Fig. 2). Overall, the indicators of the two parameters of stone burden had a high sensitivity and specificity (Fig. 2). However, in patient groups with CSD ≥20.0 mm, stone volume had a higher correlation with SF status than it did in patients with CSD <20.0 mm (Fig. 2). This must be because the formula for stone volume using NCCT with wide slice provides a more accurate value for larger stones.

On the contrary, the result of stone diameter was completely opposite to that of stone volume. In addition, Spearman’s rank-correlation coefficient values (ρ) indicated a lower correlation between the two parameters in cases with CSD ≥20.0 mm than in those with CSD <20.0 mm (Fig. 2). Table 1 revealed that there were significant differences between the <20.0 mm diameter and ≥20.0 mm diameter groups in the number of stones (P<0.001). This suggested that the parameter of stone number influenced the power of CSD as a predictor of SF status. That is, the more stones, the lower the correlation between the two parameters of stone burden. To assess our hypothesis, we evaluated the impact of stone number on the correlation between CSD and stone volume (Fig. 2). The findings shown in Fig. 2 suggested that the predictive power of CSD was equal to that of stone volume in cases where there were 1–3 stones, but in cases with ≥4 stones, CSD had a lower correlation with SF status than stone volume.

In addition, Spearman’s ρ indicated a strong correlation between the two parameters in cases with ≤3 stones (Fig. 3). In cases with ≥4 stones, however, Spearman’s ρ showed much a lower correlation (Fig. 3). Considering the rationale for using CSD, there is no doubt that in multiple stone cases, some CSDs represent smaller stone volumes, which may be responsible for the present limitation of CSD. That limitation would also account for the low correlation between the two parameters in cases with CSD ≥20.0 mm (Figs. 1, 2).

This study indicated that, in cases with CSD ≥20.0 mm or with ≥4 stones, we should evaluate stone volume as the preoperative stone burden. Moreover, NCCT is necessary at pretreatment evaluation in these cases [13]–[15]. In cases with CSD <20.0 mm or 1–3 stones, CSD remains a validated preoperative stone burden, as well as stone volume. This also suggested that the KUB films were enough to evaluate preoperative analysis of stone burden in these cases.

There are inherent limitations to this study. Because of its retrospective design, confounding factors and measurement bias cannot be reduced as much as they can in prospective, randomized studies. To try to offset this, we included procedures conducted by a large number of urologists that could influence outcome. Another limitation of the study was the involvement of only a single medical center. Additional studies from multiple centers are warranted.

### Conclusions

The findings indicated that, in cases with CSD ≥20.0 mm or ≥4 stones, we should evaluate stone volume for a more predictive stone burden. Moreover, this suggests that only the non-contrast CT scans taken at pretreatment evaluation were necessary in these cases. In cases with CSD <20.0 mm or with 1–3 stones, CSD proved to be as valid a predictor of preoperative stone burden as stone volume, which suggests that KUB films may be enough to evaluate preoperative analysis of stone burden in these cases. This data supports clinicians’ efforts to minimize radiation exposure by using KUB on all patients, so as to be selective in the use of NCCT.

## References

[1] Preminger GM, Tiselius HG, Assimos DG, Alken P, Buck C, et al (2007) EAU/AUA Nephrolithiasis Guideline Panel. 2007 guideline for the management of ureteral calculi. J Urol 178(6): 2418–34. 17993340 10.1016/j.juro.2007.09.107

[2] Tiselius HG, Ackermann D, Alken P, Buck C, Conort P, et al (2001) Working Party on Lithiasis, European Association of Urology. Guidelines on urolithiasis. Eur Urol 40(4): 362–71. 11713390 10.1159/000049803

[3] Breda A, Ogunyemi O, Leppert JT, Schulam PG (2009) Flexible ureteroscopy and laser lithotripsy for multiple unilateral intrarenal stones. Eur Urol 55(5): 1190–6. 18571315 10.1016/j.eururo.2008.06.019

[4] Riley JM, Stearman L, Troxel S (2009) Retrograde ureteroscopy for renal stones larger than 2.5 cm. J Endourol 23(9): 1395–8. 19694527 10.1089/end.2009.0391

[5] Mariani AJ (2007) Combined electrohydraulic and holmium:YAG laser ureteroscopic nephrolithotripsy of large (greater than 4 cm) renal calculi. J Urol 177(1): 168–73. 17162030 10.1016/j.juro.2006.08.066

[6] Tan PK, Tan EC, Tung KH, Foo KT (1995) Extracorporeal shock wave lithotripsy monotherapy for selected staghorn stones. Singapore Med J 36(1): 53–5. 7570136

[7] Lam HS, Lingeman JE, Barron M, Newman DM, Mosbaugh PG (1992) Staghorn calculi: analysis of treatment results between initial percutaneous nephrostolithotomy and extracorporeal shock wave lithotripsy monotherapy with reference to surface area. J Urol 147(5): 1219–25. 1569653 10.1016/s0022-5347(17)37522-5

[8] Ito H, Kawahara T, Terao H, Ogawa T, Yao M (2012) The Most Reliable Preoperative Assessment of Renal Stone Burden as a Predictor of Stone-free Status After Flexible Ureteroscopy With Holmium Laser Lithotripsy: A Single-center Experience. Urology 80(3): 524–8. 22658621 10.1016/j.urology.2012.04.001

[9] Hyams ES, Bruhn A, Lipkin M, Shah O (2010) Heterogeneity in the reporting of disease characteristics and treatment outcomes in studies evaluating treatments for nephrolithiasis. J Endourol 24(9): 1411–4. 20629563 10.1089/end.2009.0645

[10] Ito H, Kawahara T, Terao H, Ogawa T, Yao M (2012) Predictive value of attenuation coefficients measured as hounsfield units on noncontrast computed tomography during flexible ureteroscopy with holmium laser lithotripsy: a single-center experience. J Endourol 26(9): 1125–30. 22519718 10.1089/end.2012.0154

[11] Eisner BH, Kambadakone A, Monga M, Anderson JK, Thoreson AA (2009) Computerized tomography magnified bone windows are superior to standard soft tissue windows for accurate measurement of stone size: an in vitro and clinical study. J Urol 181(4): 1710–5. 19230922 10.1016/j.juro.2008.11.116

[12] Bewick V, Cheek L, Ball J (2004) Statistics review 13: Receiver operating characteristic (ROC) curves. Crit Care 8: 508–512. 15566624 10.1186/cc3000 PMC1065080

[13] Narepalem N, Sundaram CP, Boridy IC (2002) Comparison of helical computerized tomography and plain radiography for estimating urinary stone size. J Urol 167(3): 1235–8. 11832704

[14] Parsons JK, Lancini V, Shetye K (2003) Urinary stone size: comparison of abdominal plain radiography and noncontrast CT measurements. J Endourol 17(9): 725–8. 14642030 10.1089/089277903770802245

[15] Dundee P, Bouchier-Hayes D, Haxhimolla H (2006) Renal tract calculi: comparison of stone size on plain radiography and noncontrast spiral CT scan. J Endourol 20(12): 1005–9. 17206892 10.1089/end.2006.20.1005

